# Supplementary figures and images for: Functional plasticity in chromosome–microtubule coupling on the evolutionary time scale
Source: Life Sci Alliance. 2023 Oct 4;6(12):e202201720. doi: 10.26508/lsa.202201720 (PMC10551642; doi:10.26508/lsa.202201720)

anti-prot A blot

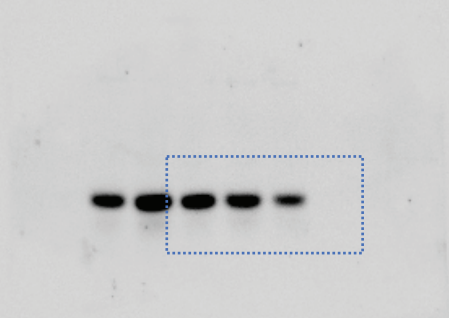

anti-PSTAIRE blot

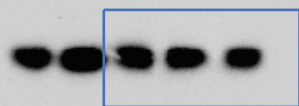

Supplement: Supplementary file 2 [file LSA-2022-01720_SdataF4.1.pdf]
