# Supplementary material for: Functional plasticity in chromosome–microtubule coupling on the evolutionary time scale
Source: Life Sci Alliance. 2023 Oct 4;6(12):e202201720. doi: 10.26508/lsa.202201720 (PMC10551642; doi:10.26508/lsa.202201720)
Supplement: Supplementary file 3 [file LSA-2022-01720_TableS1.docx]

**Table S1. Distribution of point and regional centromere structures across Eukaryota.**

| **Taxonomic position** | | **Species** | ***CEN* structure** | ***CEN* size** | **Ref** |
| --- | --- | --- | --- | --- | --- |
| Opisthokonta | Fungi (Ascomycota) | *Saccharomyces cerevisiae* | point | 125 bp | (Fitzgerald-Hayes et al. 1982) |
|  |  | *Naumovozyma castellii* | point | 125 bp | (Kobayashi et al. 2015) |
|  |  | *Naumovozyma dairenensis* | point | 125 bp | (Kobayashi et al. 2015) |
|  |  | *Saccharomyces bayanus* | point | 125 bp | (Gordon et al. 2011) |
|  |  | *Candida glabrata* | point | 125 bp | (Gordon et al. 2011) |
|  |  | *Vanderwalozyma polyspora* | point | 125 bp | (Gordon et al. 2011) |
|  |  | *Zygosaccharomyces rouxii* | point | 125 bp | (Gordon et al. 2011) |
|  |  | *Lachancea kluveri* | point | 125 bp | (Gordon et al. 2011) |
|  |  | *Ashbya gossypii* | point | 125 bp | (Gordon et al. 2011) |
|  |  | *Kluyveromyces lactis* | point | 125 bp | (Gordon et al. 2011) |
|  |  | *Candida albicans* | regional | 3-5 kb | (Sanyal et al. 2004) |
|  |  | *Candida dubliniensis* | regional | 3-5 kb | (Padmanabhan et al. 2008) |
|  |  | *Candida tropicalis* | regional | 10-18 kb | (Chatterjee et al. 2016) |
|  |  | *Candida parapsilosis* | regional | 1-2.6 kb | (Guin et al. 2020a; Ola et al. 2020) |
|  |  | *Candida viswanathii* | regional | 11-16 kb | (Guin et al. 2020a) |
|  |  | *Candida sojae* | regional | 7-26 kb | (Guin et al. 2020a) |
|  |  | *Candida auris* | regional | 2-3 kb | (Narayanan et al. 2021) |
|  |  | *Candida lusitaniae* | regional | 4-4.6 kb | (Kapoor et al. 2015) |
|  |  | *Candida haemulonii* | regional | 2-3 kb | (Narayanan et al. 2021) |
|  |  | *Candida duobushaamulonii* | regional | 2-3 kb | (Narayanan et al. 2021) |
|  |  | *Candida pseudohaemulonii* | regional | 2-3 kb | (Narayanan et al. 2021) |
|  |  | *Kuraishia capsulata* | regional | 3.5-6 kb | (Marie-Nelly et al. 2014) |
|  |  | *Ogataea polymorpha* | regional | <10 kb | (Ravin et al. 2013) |
|  |  | *Blastobotrys adeninivorans* | regional | ~6 kb | (Kunze et al. 2014) |
|  |  | *Yarrowia lipolytica* | regional | <200 bp | (Fournier et al. 1993) |
|  |  | *Komgaetaella phaffii* | regional | 3-5 kb | (Coughlan et al. 2016) |
|  |  | *Schefferosomyces. stipites* | regional | ~10 kb | (Coughlan and Wolfe 2019) |
|  |  | *Zymoseptoria tritici* | regional | 5-12.5 kb | (Schotanus et al. 2015) |
|  |  | *Neurospora crassa* | regional | 170-300 kb | (Cambareri et al. 1998) |
|  |  | *Magnaporthe oryzae* | regional | 57-109 kb | (Yadav et al. 2019) |
|  |  | *Schizosaccharomyces pombe* | regional | 35-110 kb | (Nakaseko et al. 1986; Fishel et al. 1988) |
|  | Fungi (Basidiomycota) | *Cryptococcus neoformans* | regional | 27-64 kb | (Janbon et al. 2014; Yadav et al. 2018) |
|  |  | *Cryptococcus deuterogattii* | regional | 8-22 kb | (Janbon et al. 2014; Yadav et al. 2018) |
|  |  | *Malassezia sympodialis* | regional | 3-5.5 kb | (Sankaranarayanan et al. 2020) |
|  |  | *Malassezia furfur* | regional | 3-5.5 kb | (Sankaranarayanan et al. 2020) |
|  |  | *Ustilago maydis* | regional | 7-38 kb | (Yadav et al. 2018) |
|  | Fungi (Mucoromycota) | *Mucor circinelloides* | mosaic of point and regional | ~1 kb | (Navarro-Mendoza et al. 2019) |
|  | Animals | *Drosophila melanogaster* | regional | 200-500 kb | (Sun et al. 1997) |
|  |  | Equus asinus | regional | 54-345 kb | (Nergadze et al. 2018) |
|  |  | Gallus gallus | regional | >30 kb | (Shang et al. 2010) |
|  |  | Mus musculus | regional | 300-500 kb | (Kipling et al. 1991) |
|  |  | Homo sapiens | regional | 0.3-5 Mb | (Lo et al. 2001) |
| Amoebozoa | Amoeba  (Slime mold) | Dictyostelium discoideum | regional | 171-361 kb | (Glockner and Heidel 2009) |
| Stramenopila-Alveoloata-Rhizaria  (SAR) | Stramenopiles | *Phaeodactylum tricornutum* | regional | 2-5.6 kb | (Diner et al. 2017) |
|  |  | *Phytopthora sojae* | regional | 211-356 kb | (Fang et al. 2020) |
|  | Alveolates | Plasmodium falciparum | regional | 2-2.5 kb | (Kelly et al. 2006) |
|  |  | *Toxoplasma gondii* | regional | 13-20 kb | (Brooks et al. 2011) |
| Archeaplastida | Red algae | Cyanidioschyzon merolae | regional | 1-4 kb | (Kanesaki et al. 2015) |
|  | Land plants (Angiosperms) | Oryza sativa | regional | 420-820 kb | (Yan et al. 2008) |
|  |  | *Arabidopsis thaliana* | regional | 40-148 kb | (Maluszynska and Heslop-Harrison 1991) |
|  |  | Brassica campestris | regional | 20.2-10 Mb | (Harrison and Heslop-Harrison 1995) |
|  |  | Sorghum bicolor | regional | ND | (Jiang et al. 1996) |
|  |  | Triticum aestivum | regional | ND | (Kishii et al. 2001) |
|  |  | Zea mays | regional | 2-10 Mb | (Ananiev et al. 1998) |
|  |  | Solanum tuberosum | regional | 1-3 Mb | (Gong et al. 2012) |
| Excavata | Kinetoplastid | *Trypanosoma bruceii* | regional | 20-120 kb | (Obado et al. 2007; Echeverry et al. 2012) |

ND, not determined
