# Supplementary material for: Functional plasticity in chromosome–microtubule coupling on the evolutionary time scale
Source: Life Sci Alliance. 2023 Oct 4;6(12):e202201720. doi: 10.26508/lsa.202201720 (PMC10551642; doi:10.26508/lsa.202201720)
Supplement: Supplementary file 4 [file LSA-2022-01720_TableS2.docx]

**Table S2- List of strains used in this study**

| **Strain** | **Parent** | **Genotype** |
| --- | --- | --- |
| ***S. cerevisiae strains*** | | |
| CJY077 |  | *MAT*a *Δdad2::KanMX6 ura3‐52 lys2‐801 ade2‐101 trp1Δ63 leu2Δ1::pCJ055(DAD2^TS^, LEU2) his3Δ200* [23] |
| YSR01 | CJY077 | *MAT*a *Δdad2::KanMX6 ura3‐52 lys2‐801 ade2‐101 trp1Δ63 leu2Δ1::pCJ055 his3Δ200 pRS313G (GFP, HIS3)* |
| YSR02 | CJY077 | *MAT*a *Δdad2::KanMX6 ura3‐52 lys2‐801 ade2‐101 trp1Δ63 leu2Δ1::pCJ055 his3Δ200 pSR01 (DAD2-FL-GFP, HIS3,CEN6)* |
| YSR03 | CJY077 | *MAT*a *Δdad2::KanMX6 ura3‐52 lys2‐801 ade2‐101 trp1Δ63 leu2Δ1::pCJ055 his3Δ200 pSR02 (DAD2-R126A-GFP, HIS3,CEN6)* |
| YSR04 | CJY077 | *MAT*a *Δdad2::KanMX6 ura3‐52 lys2‐801 ade2‐101 trp1Δ63 leu2Δ1::pCJ055 his3Δ200 pSR03 (DAD2-R128A-GFP, HIS3,CEN6)* |
| YSR05 | CJY077 | *MAT*a *Δdad2::KanMX6 ura3‐52 lys2‐801 ade2‐101 trp1Δ63 leu2Δ1::pCJ055 his3Δ200 pSR04 (DAD2-ΔDSS-GFP, HIS3,CEN6)* |
| BY4741 |  | MATa his3Δ1 leu2Δ0 met15Δ0 ura3Δ0 |
| YSR06 | BY4741 | MATa his3Δ1 leu2Δ0 met15Δ0 ura3Δ0 *SPC42::SPC42-mCherry-KanMX4* |
| YSR07 | YSR06 | MATa his3Δ1 leu2Δ0 met15Δ0 ura3Δ0 *SPC42::SPC42-mCherry- KanMX4* pSR05(Dad2-FL,*URA3,CEN6)* |
| YSR08 | YSR07 | MATa his3Δ1 leu2Δ0 met15Δ0 ura3Δ0 *SPC42::SPC42-mCherry- KanMX4 Δdad2::LEU2* pSR05(Dad2-FL*,URA3,CEN6)* |
| YSR09 | YSR08 | MATa his3Δ1 leu2Δ0 met15Δ0 ura3Δ0 *SPC42::SPC42-mCherry- KanMX4 Δdad2::LEU2* pSR05(Dad2 ^-^FL*,URA3,CEN6) pSR01 (DAD2-FL-GFP, HIS3,CEN6)* |
| YSR10 | YSR08 | MATa his3Δ1 leu2Δ0 met15Δ0 ura3Δ0 *SPC42::SPC42-mCherry- KanMX4 Δdad2::LEU2* pSR05(Dad2- FL*,URA3,CEN6) pSR02 (DAD2-R126A-GFP, HIS3,CEN6)* |
| YSR11 | YSR08 | MATa his3Δ1 leu2Δ0 met15Δ0 ura3Δ0 *SPC42::SPC42-mCherry- KanMX4 Δdad2::LEU2* pSR05(Dad2-FL*,URA3,CEN6) pSR03(DAD2-R128A-GFP, HIS3,CEN6)* |
| YSR12 | YSR08 | MATa his3Δ1 leu2Δ0 met15Δ0 ura3Δ0 *SPC42::SPC42-mCherry- KanMX4 Δdad2::LEU2* pSR05(Dad2-FL*,URA3,CEN6) pSR04 (DAD2-ΔDSS-GFP, HIS3,CEN6)* |
| SBY12503 |  | MAT a pCUP1-GFP12-LacI12:HIS3 CEN3::33LacO:KanMX SPC110-mCherry:hphMX HSK3-3V5-IAA7:KanMX bar1-1 ade3∆  (UMBRIET N. et al., Nat Commun, 2014; received from S. Biggins, Fred Hutch Cancer Center) |
| YSR13 | SBY12503 | MAT a pCUP1-GFP12-LacI12:HIS3 CEN3::33LacO:KanMX SPC110-mCherry:hphMX HSK3-3V5-IAA7:KanMX bar1-1 ade3∆ dad2::GAL_1-10_prDAD2 (NAT) |
| YSR14 | YSR13 | MAT a pCUP1-GFP12-LacI12:HIS3 CEN3::33LacO:KanMX SPC110-mCherry:hphMX HSK3-3V5-IAA7:KanMX bar1-1 ade3∆ dad2::GAL_1-10_prDAD2 (NAT) DAD2pr-DAD2-FL (LEU2) |
| YSR15 | YSR13 | MAT a pCUP1-GFP12-LacI12:HIS3 CEN3::33LacO:KanMX SPC110-mCherry:hphMX HSK3-3V5-IAA7:KanMX bar1-1 ade3∆ dad2::GAL_1-10_prDAD2 (NAT) DAD2pr-DAD2-R126A (LEU2) |
| YSR16 | YSR13 | MAT a pCUP1-GFP12-LacI12:HIS3 CEN3::33LacO:KanMX SPC110-mCherry:hphMX HSK3-3V5-IAA7:KanMX bar1-1 ade3∆ dad2::GAL_1-10_prDAD2 (NAT) DAD2pr-DAD2-R128A (LEU2) |
| YSR17 | YSR13 | MAT a pCUP1-GFP12-LacI12:HIS3 CEN3::33LacO:KanMX SPC110-mCherry:hphMX HSK3-3V5-IAA7:KanMX bar1-1 ade3∆ dad2::GAL_1-10_prDAD2 (NAT) DAD2pr-DAD2-ΔDSS (LEU2) |
| ***C. albicans strains*** | | |
| SN148 |  | *Δura3::imm434/Δura3::imm434,Δhis1::hisG/Δhis1::hisG Δarg4::hisG/Δarg4::hisG, Δleu2::hisG/Δleu2::hisG* (Noble and Johnson, 2005) |
| J108 |  | *Δura3::imm434/Δura3::imm434,Δhis1::hisG/Δhis1::hisG Δarg4::hisG/Δarg4::hisG dad2::HIS1/PCK1pr-DAD2 (URA3)* (Thakur and Sanyal, 2011) |
| J108A | J108 | *Δura3::imm434/Δura3::imm434,Δhis1::hisG/Δhis1::hisG Δarg4::hisG/Δarg4::hisG dad2::HIS1/PCK1pr-DAD2 (URA3) RPS1/RPS1:DAD2pr-DAD2^FL^-TAP(NAT)* |
| J108B | J108 | *Δura3::imm434/Δura3::imm434,Δhis1::hisG/Δhis1::hisG Δarg4::hisG/Δarg4::hisG dad2::HIS1/PCK1pr-DAD2 (URA3) RPS1/RPS1:DAD2pr-DAD2^ΔDSS^-TAP(NAT)* |
| ASR01 | SN148 | *Δura3::imm434/Δura3::imm434,Δhis1::hisG/Δhis1::hisG Δarg4::hisG/Δarg4::hisG, Δleu2::hisG/Δleu2::hisG dad2::HIS1/DAD2* |
| ASR02  (CaDad2-FL) | ASR01 | *Δura3::imm434/Δura3::imm434,Δhis1::hisG/Δhis1::hisG Δarg4::hisG/Δarg4::hisG, Δleu2::hisG/Δleu2::hisG dad2::HIS1/DAD2-FL-GFP(URA3)* |
| ASR03  (CaDad2-R92A) | ASR01 | *Δura3::imm434/Δura3::imm434,Δhis1::hisG/Δhis1::hisG Δarg4::hisG/Δarg4::hisG, Δleu2::hisG/Δleu2::hisG dad2::HIS1/DAD2-R92A-GFP(URA3)* |
| ASR04  (CaDad2-ΔDSS) | ASR01 | *Δura3::imm434/Δura3::imm434,Δhis1::hisG/Δhis1::hisG Δarg4::hisG/Δarg4::hisG, Δleu2::hisG/Δleu2::hisG dad2::HIS1/DAD2-ΔDSS-GFP(URA3)* |
| ASR07  (CaDad2-FL,CENP-A-TAP) | ASR02 | *Δura3::imm434/Δura3::imm434,Δhis1::hisG/Δhis1::hisG Δarg4::hisG/Δarg4::hisG, Δleu2::hisG/Δleu2::hisG dad2::HIS1/DAD2-FL-GFP(URA3) CSE4/CSE4-TAP (LEU2)* |
| ASR08  (CaDad2-R92A, CENP-A-TAP) | ASR03 | *Δura3::imm434/Δura3::imm434,Δhis1::hisG/Δhis1::hisG Δarg4::hisG/Δarg4::hisG, Δleu2::hisG/Δleu2::hisG dad2::HIS1/DAD2-R92A-GFP(URA3)* |
| ASR09  (CaDad2-ΔDSS, CENP-A-TAP) | ASR04 | *Δura3::imm434/Δura3::imm434,Δhis1::hisG/Δhis1::hisG Δarg4::hisG/Δarg4::hisG, Δleu2::hisG/Δleu2::hisG dad2::HIS1/DAD2-ΔDSS-GFP(URA3) CSE4/CSE4-TAP (LEU2)* |
| ASR12  (CaDad2-FL, Tub4-mCherry) | ASR02 | *Δura3::imm434/Δura3::imm434,Δhis1::hisG/Δhis1::hisG Δarg4::hisG/Δarg4::hisG, Δleu2::hisG/Δleu2::hisG dad2::HIS1/DAD2-FL-GFP(URA3) TUB4/TUB4-mCherry(NAT)* |
| ASR13  (CaDad2-R92A, Tub4-mCherry) | ASR03 | *Δura3::imm434/Δura3::imm434,Δhis1::hisG/Δhis1::hisG Δarg4::hisG/Δarg4::hisG, Δleu2::hisG/Δleu2::hisG dad2::HIS1/DAD2-R92A-GFP(URA3) TUB4/TUB4-mCherry(NAT)* |
| ASR14  (CaDad2-ΔDSS, Tub4-mCherry) | ASR04 | *Δura3::imm434/Δura3::imm434,Δhis1::hisG/Δhis1::hisG Δarg4::hisG/Δarg4::hisG, Δleu2::hisG/Δleu2::hisG dad2::HIS1/DAD2-ΔDSS-GFP(URA3) TUB4/TUB4-mCherry(NAT)* |
| ASR03M | ASR03 | *Δura3::imm434/Δura3::imm434,Δhis1::hisG/Δhis1::hisG Δarg4::hisG/Δarg4::hisG, Δleu2::hisG/Δleu2::hisG dad2::HIS1/DAD2-R92A-GFP(URA3) mad2::LEU2/MAD2* |
| ASR17  (CaDad2-R92A, *mad2*) | ASR03M | *Δura3::imm434/Δura3::imm434,Δhis1::hisG/Δhis1::hisG Δarg4::hisG/Δarg4::hisG, Δleu2::hisG/Δleu2::hisG dad2::HIS1/DAD2-R92A-GFP(URA3) mad2::LEU2/mad2::ARG4* |
| ASR04M | ASR04 | *Δura3::imm434/Δura3::imm434,Δhis1::hisG/Δhis1::hisG Δarg4::hisG/Δarg4::hisG, Δleu2::hisG/Δleu2::hisG dad2::HIS1/DAD2-ΔDSS-GFP(URA3) mad2::LEU2/MAD2* |
| ASR18  (CaDad2-ΔDSS, *mad2*) | ASR04M | *Δura3::imm434/Δura3::imm434,Δhis1::hisG/Δhis1::hisG Δarg4::hisG/Δarg4::hisG, Δleu2::hisG/Δleu2::hisG dad2::HIS1/DAD2-ΔDSS-GFP(URA3) mad2::LEU2/mad2::ARG4* |
| ASR19  (CaDad2-FL, Ndc80-mCherry) | ASR02 | *Δura3::imm434/Δura3::imm434,Δhis1::hisG/Δhis1::hisG Δarg4::hisG/Δarg4::hisG, Δleu2::hisG/Δleu2::hisG dad2::HIS1/DAD2-FL-GFP(URA3) NDC80/NDC80-mCherry-ARG4* |
| ASR20  (CaDad2-R92A, Ndc80-mCherry) | ASR03 | *Δura3::imm434/Δura3::imm434,Δhis1::hisG/Δhis1::hisG Δarg4::hisG/Δarg4::hisG, Δleu2::hisG/Δleu2::hisG dad2::HIS1/DAD2-R92A-GFP(URA3) NDC80/NDC80-mCherry-ARG4* |
| ASR21  (CaDad2-ΔDSS, Ndc80-mCherry) | ASR04 | *Δura3::imm434/Δura3::imm434,Δhis1::hisG/Δhis1::hisG Δarg4::hisG/Δarg4::hisG, Δleu2::hisG/Δleu2::hisG dad2::HIS1/DAD2-ΔDSS-GFP(URA3) NDC80/NDC80-mCherry-ARG4* |
| ***C. neoformans strains*** | | |
| CNVY108 |  | *α H99::GFP-H4-NAT (pVY3)* (Kozubowski et al., mBio, 2013) |
| SHR741 |  | *α H99::GFP-H4-NAT, mad2::NEO (Sridhar et al., Nat. Commun., 2021)* |
| CNSD169 | CNVY108 | *α H99::GFP-H4-NAT DAD2::DAD2p-DAD2-mCherry-NEO* |
| CNSD170 | CNVY108 | *α H99::GFP-H4-NAT DAD2::DAD2p-DAD2-R102A-mCherry-NEO* |
| CNSD171 | CNVY108 | *α H99::GFP-H4-NAT DAD2::DAD2p-DAD2-ΔDSS-mCherry-NEO* |
| CNVY104 |  | *α H99::GFP-DAD1-NAT (pVY2)* (Kozubowski et al., mBio, 2013) |
| CNSD198 | CNSD169 | *α H99::GFP-DAD1-NAT (pVY2) DAD2::DAD2p-DAD2 -mCherry-NEO* |
| CNSD199 | CNSD170 | *α H99::GFP-DAD1-NAT (pVY2) DAD2::DAD2p-DAD2-R102A-mCherry-NEO* |
| CNSD200 | CNSD171 | *α H99::GFP-DAD1-NAT (pVY2) DAD2::DAD2p-DAD2-ΔDSS-mCherry-NEO* |
|  |  |  |
| ***S. pombe strains*** | | |
| SP22 |  | *h- ade6-M210 leu1 lys1 ura4 cen2(D107)::KanR -ura4-lacO his7+::lacI-GFP (received from A. Marston, University of Edinburgh)* |
| SP1619 | SP22 | *h- ade6-M210 leu1 lys1 ura4 cen2(D107)::KanR -ura4-lacO his7+::lacI-GFP dad2 ::HPH (received from A. Marston, University of Edinburgh)* |
| PSR01 | SP1619 | *h- ade6-M210 leu1 lys1 ura4 cen2(D107)::KanR -ura4-lacO his7+::lacI-GFP dad2 ::HPH DAD2pr::DAD2pr-DAD2-FL-FLAG-NAT* |
| PSR02 | SP1619 | *h- ade6-M210 leu1 lys1 ura4 cen2(D107)::KanR -ura4-lacO his7+::lacI-GFP dad2 ::HPH DAD2pr::DAD2pr-DAD2-ΔDSS-FLAG-NAT* |
