# Supplementary material for: Functional plasticity in chromosome–microtubule coupling on the evolutionary time scale
Source: Life Sci Alliance. 2023 Oct 4;6(12):e202201720. doi: 10.26508/lsa.202201720 (PMC10551642; doi:10.26508/lsa.202201720)
Supplement: Supplementary file 5 [file LSA-2022-01720_TableS3.docx]

**Table S3- List of plasmids used in this study**

| **Plasmid** | **Parent** | **Description** |
| --- | --- | --- |
| pRS313 |  | CEN6/ARS/HIS3 plasmid |
| pRS313G | pRS313 | GFP along with CYC1 terminator cloned into pRS313 in BamHI-ClaI sites |
| pSR01 | pRS313G | ScDAD2-FL cloned in pRS313G in SacII-BamHI sites |
| pSR02 | pRS313G | ScDAD2-R126A cloned in pRS313G in SacII-BamHI sites |
| pSR03 | pRS313G | ScDAD2-R128A cloned in pRS313G in SacII-BamHI sites |
| pSR04 | pRS313G | ScDAD2-ΔDSS cloned in pRS313G in SacII-BamHI sites |
| pRS316 |  | CEN6/ARS/URA3 plasmid |
| pSR05 | pRS316 | ScDAD2-FL with native promoter and terminator cloned in pRS316 in SacII-SacI sites |
| pYM-N25 |  | Plasmid used to amplify cassette to place DAD2 under GAL_1-10_ promoter with NAT marker |
| pUG73 |  | Plasmid to amplify DAD2 deletion cassette with LEU2 marker, also used to generate reintegration plasmids |
| pSR06 | pUG73 | ScDAD2-FL cloned in SacII-SacI sites |
| pSR07 | pUG73 | ScDAD2-R126A cloned in SacII-SacI sites |
| pSR08 | pUG73 | ScDAD2-R128A cloned in SacII-SacI sites |
| pSR09 | pUG73 | ScDAD2-ΔDSS cloned in SacII-SacI sites |
| pBS-RN | pBS-NAT | CaRPS1 cloned in pBS NAT plasmid in NotI site |
| pRN-Dad2^FL^ | pBS-RN | CaDAD2pr-CaDAD2-FL-TAP cloned in SalI site |
| pRN-Dad2^ΔDSS^ | pBS-RN | CaDAD2pr-CaDAD2-ΔDSS -TAP cloned in SalI site |
| pRN-92 | pBS-RN | CaDAD2pr-CaDAD2-R92A-TAP cloned in SalI site |
| pCse4TAPLeu |  | CSE4-TAP with LEU2 marker (Varshney and Sanyal, 2019) |
| pTub4-mCherryNAT | pDam1-mCherryNAT | TUB4 ORF cloned in pDam1-mCherryNAT in SacII-SpeI sites |
| pMad2-2 |  | MAD2 deletion cassette with LEU2 (Thakur and Sanyal, 2011) |
| pMad2-3 |  | MAD2 deletion cassette with ARG4 (Thakur and Sanyal, 2011) |
| pSR10 | pBS-GFPUra | 3’UTR of CaDAD2 cloned in pBS-GFPUra in XhoI-KpnI sites |
| pSR11 (CaDad2-FL) | pSR10 | CaDAD2-FL_­_ ­­cloned in pSR10 in SacII-SpeI sites |
| pSR12 (CaDad2-R’A) | pSR10 | CaDAD2-R92A_­_ ­­cloned in pSR10 in SacII-SpeI sites |
| pSR15 (CaDad2-ΔDSS) | pSR10 | CaDAD2-ΔDSS ­ ­­cloned in pSR10 in SacII-SpeI sites |
| pLK25 |  | Amplification of mCherry-Neomycin for C. neoformans |
| PC4-Dad1H |  | Polycistronic vector for the expression and purification of Dam1complex from E. coli. (Miranda et al., 2005, procured from addgene) |
| PC4-126A | PC4-Dad1H | Dad2 ORF replaced to express Dad2-R126A, to purify the mutant Dam1 complex |
| PC4-ΔDSS | PC4-Dad1H | Dad2 ORF replaced to express Dad2-ΔDSS, to purify the mutant Dam1 complex |
| pVB128 |  | Yeast integrative plasmid for 3xFLAG tagging at the C terminus using NAT marker (received from V. Borde lab, Institut Curie) |
| pSp-FL | pVB128 | SpDad2pr-Dad2-FL cloned in frame with 3xFLAG for reintegration into the Dad2 promoter sequence by NheI digestion |
| pSp-ΔDSS | pVB128 | SpDad2pr-Dad2-ΔDSS cloned in frame with 3xFLAG for reintegration into the Dad2 promoter sequence by NheI digestion |
