# Supplementary material for: Functional plasticity in chromosome–microtubule coupling on the evolutionary time scale
Source: Life Sci Alliance. 2023 Oct 4;6(12):e202201720. doi: 10.26508/lsa.202201720 (PMC10551642; doi:10.26508/lsa.202201720)
Supplement: Supplementary file 6 [file LSA-2022-01720_TableS4.docx]

**Table S4- List of primers used in this study**

| ***S. cerevisiae*** | | |
| --- | --- | --- |
| ScGFP-F | CATGGATCCATGGTGAGCAAGGGCG | Cloning GFP into pRS313 |
| ScGFP-R | GCTGATCGATGCAAATTAAAGCCTTCGAGC |  |
| SR282 | TCCCGCGGCATTGCGGCAGGTAAAATATC | Amplification of Dad2^FL/MUT^ as SacII/BamHI fragment to clone into pRS313G |
| ScDad2R | GAGGGATCCTTCGTTACCATCTACCCTAATTCTG |  |
| Sc126A-R | GAGGGATCCTTCGTTACCATCTACCCTAATTGCGACCATTGTTTCC |  |
| Sc128A-R | GAGGGATCCTTCGTTACCATCTACGGCAATTCTGACCATTGTTTCC |  |
| ScΔDSS-R | GAGGGATCCTTCGTTACCATCCAAGGGTACCAGATCTTC |  |
| ScDad2Pr F | TGACCGCGGGTACAATGGTCCTAACTTAATG | Cloning ScDad2-FL into pRS316 |
| ScDad2R | ATCGAGCTCCCAACACTGTAGAATACTAATATC |  |
| ScDad2delF | GAATATATCTAAAAAACTATTGAATAGGTTTGAAAAACTCATAATTCAGACAGTTATTGCTGTGAAGATCCCAGCAAAGG | Deletion of genomic allele of DAD2 with LEU2 marker |
| ScDad2delR | ATGTATATGCTATTATCGCAACTGCCTTCTTCCGATTTATATAAGATCCTTTTCTTTCTGTGCAGGCTAACCGGAACCTGT |  |
| ScDad2F | GGTTAGAGGGCGACAATAC | Confirmatory primers |
| Leu2R | CACCAGTGTTCAACTCAACAAG |  |
| GalDad2FP | CTAAAAAACTATTGAATAGGTTTGAAAAACTCATAATTCAGACAGTTATTCGACATGGAGGCCCAGAATAC | Cassette to place DAD2 under GAL promoter with NAT marker |
| GalDad2RP | GACTGAAGTTCTTTTCGCTTTATAGCAATTTGTTCATCTATTGAATCCATTTTGTACAATTCATCCATACCATGG |  |
| Dad2pr-SacII-F | TGACCGCGGGTACAATGGTCCTAACTTAATG | Overlap PCR primers to amplify DAD2^FL/MUT^ for cloning into pUG73 for reintegration |
| Dad2pr-SacI-R | ATCGAGCTCCCAACACTGTAGAATACTAATATC |  |
| ScTer FP | GATGGTAACGAATGAACAGAAAG |  |
| Sc126A-R | CTTTCTGTTCATTCGTTACCATCTACCCTAATTGCGACCATTGTTTCC |  |
| Sc128A-R | CTTTCTGTTCATTCGTTACCATCTACGGCAATTCTGACCATTGTTTCC |  |
| ScΔDSS-R | CTTTCTGTTCATTCGTTACCCAAGGGTACCAGATCTTC |  |
| ***C. albicans*** | | |
| RP10F | ATAAGAATGCGGCCGCTAGATCCAACTCAAGTAC  AACATGGC | amplification and cloning of RPS1 locus into pBS-NAT |
| RP10R | ATAAGAATGCGGCCGCGGATCCCCCAGATCATTA  TCC |  |
| AD02 | ACGCGTCGACATTTTGACTAGT  TCTCAAATGGTTC | Overlap PCR primers to amplify CaDAD2^FL/MUT^ and clone into pBS-RP10-NAT |
| AD03 | CCATCGATCGATATCAAGCTTCAGGTTG |  |
| Dad2delR | GTCTTGATTTTCTTCATTTGATTGTCCGAAAGCCTCTTTGTTATTATC |  |
| Dad2delF | GATAATAACAAAGAGGCTTTCGGACAATCAAATGAAGAAAATCAAGAC |  |
| R92A FP | GAACCATTAGTAGCAGTGCGTGTTGGACAATCA |  |
| R92A RP | TGATTGTCCAACACGCACTGCTACTAATGGTTC |  |
| Dad2DS-F | GGCCTCGAGGTGTACATATAATAACTCTAAATTCTGGC | Amplification of Dad2 3’UTR homology |
| Dad2DS-R | ACCGGTACCGAATTTTGTCAACCAAGAAATAGAC |  |
| Dad2FP | TACCCGCGGATGCTGAAAACAAATACTGCTATATACC | Amplification of DAD2 ORF tagging with GFP |
| Dad2GFP-RP | CGACTAGTTTCCGTGGATTCTTCAACTTC |  |
| Dad2cFP | TCAATACCCACCACAAAACC | Confirmatory primer |
| ***C. neoformans*** | | |
| SD118 | TGCATGCATTCTCGTCAAAATAGGCTGC | Overlap PCR primers to amplify CnDAD2^FL/R102A^ |
| SD119 | TCGCCGCCGTATGCTAAGGCCACGAGACAAGGGAGAGG |  |
| SD120 | CCTCTCCCTTGTCTCGTGGCCTTAGCATACGGCGGCGAA |  |
| VYP152 | CTCGCCCTTGCTCACCATTTGTTGTTTTGTTTTATCAGATGCG |  |
| VYP153 | CTGATAAAACAAAACAACAAATGGTGAGCAAGGGCGAG | Amplification of mCherry-NEO from pLK25 |
| VYP154 | CTATTGGTCGTCATCAGCAGGCCAAGCTTGGTACCGAGCTC |  |
| VYP155 | GAGCTCGGTACCAAGCTTGGCCTGCTGATGACGACCAATAG | amplification CnDAD2 3’UTR homology |
| VYP156 | CCCAAGCTTCTCCATATCGTGTCTCAATTTCATCTC |  |
| VYP157 | CTTGTACAGCTCGTCCATGC | Confirmatory primer |
| ***S. pombe*** | | |
| VB128 FP | CACCACCATCATCATCACGG | Amplification of the vector pVB128 |
| VB128 RP | CACCACCATCATCATCACGG |  |
| SpFP | CGGCCAGTGAATTGTAATACGACTCGGAACGTTGACAATCTTGTTG | Sp-FP along with each of the reverse primer to amplify SpDad2-FL and SpDad2-ΔDSS |
| Sp-FL-RP | ccgtgatgatgatggtggtgTACCTCTTCAACATCGCCTTGC |  |
| Sp-ΔDSS-RP | ccgtgatgatgatggtggtgTACCTCTTCAACATCGCCTTGCTCCGTAGCAGATGCGTTGGTATTTGAAGTATGTTGGGATGCTATCTGTATTGAC |  |
| Sp-cFP | GGTATTCTTTAACGACCCGTTG | Primers to confirm targeted integration |
| FLAG-cRP | GAGGCAAGCTAAACAgatcTC |  |
